# Public deliberation on health gain measures

**DOI:** 10.1093/haschl/qxae111

**Published:** 2024-09-09

**Authors:** Ching-Hsuan Lin, Tara A Lavelle, Marie C Phillips, Abigail G Riley, Daniel Ollendorf

**Affiliations:** Center for the Evaluation of Value and Risk in Health, Tufts Medical Center, 800 Washington St, Boston, MA 02111, United States; Center for the Evaluation of Value and Risk in Health, Tufts Medical Center, 800 Washington St, Boston, MA 02111, United States; Center for the Evaluation of Value and Risk in Health, Tufts Medical Center, 800 Washington St, Boston, MA 02111, United States; Center for the Evaluation of Value and Risk in Health, Tufts Medical Center, 800 Washington St, Boston, MA 02111, United States; Center for the Evaluation of Value and Risk in Health, Tufts Medical Center, 800 Washington St, Boston, MA 02111, United States

**Keywords:** public deliberation, rank, health gain measure, quality-adjusted life-year (QALY), clinical benefit rating, multicriteria decision analysis (MCDA), dashboard

## Abstract

Researchers and decision-makers use health gain measures to assess the value of health interventions. However, our current understanding of how these measures are understandable and accessible to the community is limited. This study examined a diverse group of stakeholders’ attitudes and preferences for 9 commonly used health gain measures. We recruited 20 stakeholders, including patients, caregivers, pharmacists, allied health professionals, and citizens. We conducted 2 in-person deliberative meetings in which participants learned, discussed, deliberated on, and ranked 9 health gain measures. The final ranking conducted after unified deliberation showed the quality-adjusted life year (QALY) as the top-ranked measure, followed by the clinical benefit rating method used by the U.S. Preventive Services Task Force, and multicriteria decision analysis (MCDA). We identified 3 themes during deliberations: the importance of using patient values in population-based health gain measures, examining complementary measures together, and choosing measures that are intuitive and easy to understand. Future policymaking should consider incorporating the QALY, clinical benefit rating, and MCDA into prioritization decisions.

## Introduction

There is no universally agreed upon measure for summarizing health benefits, and “health gain” measures may differ in terms of how they present outcomes. These measures aim to evaluate the value of an intervention. They may be presented alongside other outcome measures, including economic outcomes such as those in a health technology assessment (HTA), cost-effectiveness analysis (CEA), or benefit–cost analysis.^1^ They may also facilitate policy discussions and patient care.

Some health gain measures have been the subject of controversy and heated debate, most recently in the context of the Inflation Reduction Act (IRA) of 2022. The IRA authorizes the Secretary of the Department of Health and Human Services to negotiate drug prices for the Medicare program, the single largest purchaser of prescription drugs in the United States. Notably, it has prohibited the use of quality-adjusted life years (QALYs), a health gain measure endorsed by the US Panel on Cost-effectiveness in Health and Medicine, in negotiations.^2,3^ This is largely due to concerns that the QALY might discriminate against disabled individuals because they have less potential to accrue QALY gains compared to younger and healthier individuals. To mitigate this issue, several methods have been proposed to modify the QALY, such as health years in total (HYT) and the equal value life year (evLY), which use different approaches to consider quality of life improvements equally across disease states.^4,5^ However, while alternative health gain measures attempt to address issues around the QALY, they also impose their own measurement concerns, and the debate on the advantages and disadvantages of each continues. Health gain measures also have applications beyond Medicare negotiations. The Institute for Clinical and Economic Review (ICER), an independent nonprofit group that aims to influence drug pricing in the United States, has generated value assessment evidence using QALY- and evLY-based methods.^4^

Despite the widespread use of health gain measures and their potential importance in drug pricing and policy decisions, there is limited documentation of public values and preferences for these measures. Discussions on the advantages and disadvantages of health gain measures have mainly occurred between policymakers and health service researchers. However, it is critical to understand not only the opinions of those using health gain measures in decision-making but also those who are affected by such decisions. Previous studies have not recruited stakeholders from diverse backgrounds to exchange viewpoints on health gain measures. Broader insights from a diverse group of stakeholders may allow for a more complete consideration of how best to communicate resource allocation decisions to the individuals affected by them.

This study investigated community stakeholders’ attitudes toward health gain measures. We conducted a public deliberation, a method that gathers informed input on complex healthcare issues affecting broad public constituencies.^5^ We asked community stakeholders (patients, caregivers, healthcare providers, and citizen representatives) to discuss and rank a range of health gain measures according to their preferences. Additionally, we engaged with stakeholders to develop a user-friendly “dashboard” tool that can display multifaceted information on the health gains that may be realized from a given treatment.

## Methods

Our public deliberation integrated the 3 elements outlined by Blacksher et al.,^6^ including the provision of information, the inclusion of diverse perspectives, and the opportunity to reflect on and discuss competing viewpoints. We also followed the recommendations of Carman et al.^7^ in the deliberation process, including procedures for recruitment, session length, use of experts, and measurement and polling.

### Outreach and recruitment

We recruited study participants using social media platforms, Craigslist, contact letters, and emails to community groups within the greater Boston area and targeted referrals from the Center for Community-Engaged Medicine at Tufts Medical Center. The goal was to recruit 30 individuals from the Boston metropolitan area, representing 5 different perspectives: patients (persons living with a medical condition), caregivers (individuals who care for a person living with a medical condition), citizens (members of the general public or representatives from a civil society group such as labor unions and professional associations), clinicians, and pharmacists or other allied health professionals. Individuals who contacted us based on our outreach were provided with study details and an online screening survey link. The screening survey asked individuals for the perspective they represented, contact information, social demographics, education level, English proficiency, numeric literacy,^5^ experience with or knowledge of approaches used to value improvements in health, and preferred meeting times for deliberation (see Appendix S1).

We recruited participants between June and September 2023. A total of 93 individuals completed the screening survey. We excluded individuals with extensive knowledge of health gain measures (*n* = 1) as well as those unable to attend in-person deliberations (*n* = 2). Among the remaining eligible participants, we selected 30 with the goals of maximizing the representation of all perspectives and ensuring demographic diversity.

Following the recommendations of Carman et al.^7^ for providing accessible educational materials, we worked with the Tufts Health Science Institutional Review Board to develop the materials at an eighth-grade reading level. We asked selected participants to review them before the first meeting, which required approximately 2 h of their time (see Appendix S2). We encouraged participants to reach out or schedule a virtual meeting if they had questions regarding the materials before attending the first in-person meeting.

Participants received travel reimbursement and $1000 for their time through the ClinCard® system (Greenphire, Inc., King of Prussia, Pennsylvania). The Tufts Health Sciences Institutional Review Board approved this study (STUDY00003719).

### Health gain measures

The stakeholders reviewed 9 commonly used health gain measures, including the QALY, disability-adjusted life year (DALY), life year (LY), evLY, HYT, added benefit, clinical benefit rating, multicriteria decision analysis (MCDA), and social return on investment (SROI). The QALY has been the de facto standard for measuring health gains in CEA for decades, and it considered both life extension (quantity) and quality of that extended life.^8^ The DALY was introduced as part of the Global Burden of Disease Study in the 1990s as a measure of the impact of disability and premature mortality on disease trajectory.^9^ As mentioned above, both HYT and evLY are tools derived from the QALY with the goal of overcoming its perceived limitations. Added benefit, introduced by the German government in The Pharmaceutical Market Restructuring Act of 2011, classifies health gains into 6 categories, ranging from “major added benefit” to “less benefit.”^10^ The clinical benefit rating, commonly used by the U.S. Preventive Services Task Force (USPSTF) and other organizations focused on clinical data synthesis, also categorizes incremental health benefits but adds another axis focused on the reviewer's level of confidence or certainty in the evidence base.^11^ The MCDA, which has been used in health and nonhealth sectors alike since the 1960s, involves assigning values to multiple quantitative and qualitative criteria and aggregating weighted values to generate a single score that reflects preference.^12^ Although MCDA is not a traditional health gain measure, we included it in our study because it can measure and weight health along with factors that are deemed important, beyond health. In addition, MCDA is increasingly used as a deliberation tool by HTA bodies.^13^ Finally, SROI incorporates broader concepts of values beyond individuals or communities, such as environmental and societal impacts, in monetary terms.^14^

### Event structure

The study consisted of 2 in-person deliberative meetings in Boston, with approximately 1 month between sessions. The first meeting served 2 primary purposes: first, to conduct a ranking exercise with input from a variety of stakeholders to identify health gain measures that may be most beneficial in common practice and, second, to summarize discussions throughout the meetings and conduct a thematic analysis to provide insight for why stakeholders ranked health gain measures in a particular way. After the first meeting, we created a dashboard based on the most highly ranked health gain measures. In the second meeting, we sought stakeholder feedback on our dashboard, aiming to make it understandable for laypeople while providing sufficient information. The first full-day meeting took place in September, followed by the second half-day meeting in October 2023.

During the morning of the first meeting, 2 faculty members from Tufts Medical Center provided a 4-h educational session on health gain measures. Based on recommendations for public deliberation, we provided balanced information by presenting ethical considerations, known controversies, and criticisms for each measure.^7^ We also allowed sufficient time for participants to ask any questions they had following each presentation and before they began deliberating. Five sessions followed the educational component. In the first session, we divided the participants into 2 breakout rooms to deliberate their perspectives and opinions on each of the health gain measures. By design, 1 room included participants identifying as patients and caregivers, and the other room included citizens, clinicians, and pharmacists or allied health professionals. Each room had 1 faculty member serving as a facilitator. Facilitators asked each participant to share their perspectives on the benefits and drawbacks of health gain measures. During the deliberations, we asked participants to discuss and exchange reasons for their preferences. When competing arguments arose, facilitators encouraged participants to elaborate and challenge each other's views. We took notes and recorded their deliberations to include them in our thematic analysis. In the second session, conducted in the same breakout configuration, each participant anonymously ranked their own preferences for the health gain measures discussed, from the most to least favorable. We used Poll Everywhere® (Poll Everywhere, San Francisco, CA), a web-based real-time audience polling system, to conduct the ranking exercise. In the third session, participants returned to a single, large convening. We presented the aggregated ranking results from each breakout room to the entire group. We had a second deliberation that encouraged participants to review, justify, and discuss their rankings. Discussions from the second deliberation were recorded and incorporated into our thematic analysis. In the fourth session, we conducted a second anonymous ranking exercise using Poll Everywhere® as a combined group. We presented the aggregated results and discussed the shifts from the first to second ranking. In the fifth session, we concluded the first in-person meeting with examples and discussion of how to visualize health gain measures as a “dashboard.”

Members of the study team recorded both meetings (with permission from participants) and supplemented recordings with written notes.

### Thematic analysis

We analyzed transcripts from deliberative discussions using thematic analysis. Prior to analysis, we identified a priori or deductive codes through team discussion of the breakout room exchanges that we observed. We then created a preliminary codebook with a priori codes. Utilizing the preliminary codebook, 2 researchers independently coded the transcripts from both breakout rooms. Next, the researchers met to compare and revise codes, and add inductive codes until they reached coding consensus. Resulting code definitions and interview example quotes were used to create a final codebook that both researchers again applied to each breakout room discussion and compared codes together until they reached consensus. Using Microsoft Word® (Microsoft, Inc., Redmond, WA), we categorized codes to major and minor themes based on the frequency of quotes identified for each code. Additionally, we summarized the quotes for each theme.

### Dashboard development

After the first meeting, we developed dashboard drafts from the top 3 health gain measures selected by the group in the final ranking exercise. We referenced publicly available information to help us develop 2 versions of dashboard drafts.^15,16^ We limited our dashboard to 3 measures for ease of visualization and interpretation. We used data from a recent ICER report on resmetirom, a drug for nonalcoholic steatohepatitis (NASH), to illustrate information for this dashboard.^17^ When data were unavailable from the ICER report, we used hypothetical values in the dashboard. We collected participants’ feedback on the draft dashboard via e-mail and revised the dashboard before the second meeting. During the second in-person meeting, participants reviewed, deliberated, and discussed how to further refine and improve the draft dashboard. We made a final revision to the dashboard after integrating all feedback from the second meeting.

## Results

Among the 30 selected participants, 27 confirmed their participation. During the week of the first in-person deliberation, 1 declined to participate after reviewing the educational materials, 4 notified us that they were unable to participate due to time conflicts or emergency issues, and 2 did not show up without any notice. A total of 20 participants took part in the deliberations, including patients and patient representatives (*n* = 8), caregivers and caregiver representatives (*n* = 4), pharmacists or allied health professionals (*n* = 4), and citizens (*n* = 4). Notably, 2 participants were clinicians, but they self-identified as a caregiver and a patient representative, respectively. Participants represented a diverse range of stakeholders across various ages, races, education, and income levels (Appendix S3). The majority of participants were White (*n* = 11) and held graduate degrees (*n* = 12). Within each stakeholder group, we recruited a diversity of perspectives with regard to age, sex, race, education, and income.

### Ranking exercise

Table 1 reports the ranking results. In the breakout sessions, the patient and caregiver group ranked the QALY highest, followed by clinical benefit rating and the evLY. The citizen and pharmacist or allied health professional group ranked MCDA highest, followed by the QALY and clinical benefit rating. Our final ranking as a large group resulted in the QALY as the most favored health gain measure, followed by clinical benefit rating and MCDA.

**Table 1.** Ranking results.

	Patients and caregivers	Citizens, pharmacists, or allied health professionals	Final ranking
QALY (quality-adjusted life year)	1	2	1
Clinical benefit rating	2	3	2
MCDA (multicriteria decision analysis)	6	1	3
Added benefit	4	5	4
SROI (social return on investment)	9	4	5
DALY (disability-adjusted life year)	5	8	6
HYT (health years in total)	8	6	7
evLY (equal value life years)	3	7	8
LY (life year)	7	9	9

### Thematic analysis

Table 2 summarizes the major themes and selected quotes by groups in breakout deliberations. There were 3 major themes captured, as described in further detail below.

1. Patient input is important for deriving values in population-based health gain measures
  Patients and caregivers highlighted that individuals with a lived experience of a particular illness understand their health condition, including symptoms and treatment effects, better than those without the health condition. These participants discussed how common perceptions that people living with an illness or caring for someone with an illness have impaired quality of life are often inaccurate. Consequently, they emphasized that health gain measures may be biased when values are derived from individuals who lack personal experience with their health state. Conversely, involving individuals who have experienced the illness in creating and summarizing health gain measures improves their accuracy. Citizens, as well as pharmacists and allied health professionals, also talked about including those affected by an illness in measurement development. To support this argument, a healthcare provider shared a story describing how patients prefer making their own decisions rather than having others decide for them.
2. Need multiple complementary measures to see full range of societal benefits
  The patient and caregiver group discussed the importance of juxtaposing different health gain measures to illustrate a comprehensive understanding of an intervention's benefits. This approach allows for a clearer assessment of the overall impact and facilitates informed decision-making. The citizen and pharmacist or allied health professional group discussed the limitations of individual health gain measures. For instance, if decision-makers solely rely on measures focusing on LYs gained—important for interventions that reduce mortality—these measures may not reflect other important benefits of an intervention, such as promoting health equity or addressing social benefits. Moreover, this group highlighted that these health gain measures are used for societal resource allocation decisions, impacting not only patients and caregivers but also other members of society. Consequently, they argued that using multiple measures to inform decisions about the selection of optimal health interventions would be advantageous and promote policy decisions that maximize societal benefits.
3. Information should be accessible, simple, and understandable to the public
  Both groups emphasized that the health gain measures should be easy to understand and apply to a broader audience. To achieve this, some participants preferred health gain measures with precise quantitative data, while others favored health gains that were classified within broad categorizations of benefit. By prioritizing simplicity and clarity, researchers and policymakers can effectively convey the meaning of the health gain measures and facilitate their application. Additionally, one participant commented that the process of developing health gain measures should be accessible to promote transparency and accountability.

**Table 2.** Selected quotes from the major themes.

Patient input is important for deriving values in population-based health gain measures.
“I wouldn't trust somebody who never had any experience with disabilities or has never been disabled to be able to weigh those things (health gain measures) (in a way) that I would feel comfortable with.”—patient/caregiver group “I went through this (disconnect between patient and community values) towards my mom's later part of life. She was bed-bound. And for some people, they used to be like, what type of life is bed-bound? But for her, it was still being able to see her grandkids, still being able to laugh, have fun, and eat. Nothing else stopped besides the fact that she couldn't walk. So, for a lot of people, that's not a good life, but for her, that's a good life.”—patient/caregiver group “The ones that use the patient input are slightly better because they're [the patients are] deciding for themselves the impact on their own lives, which personally feels much better.”—citizen/pharmacist or allied health professionals group “It feels so important to have like the actual people whose actual lives are going to be impacted, involved in the decision making on a really high level”—citizen/pharmacist or allied health professionals group

Need multiple complementary measures to see full range of societal benefits.
“What I find frustrating about it is when I first decided to do this, I thought about health gains. Most of the measurements are focused on patients, and the patients’ immediate surrounding in their care group is their family. But if you're thinking about policy and how much we should be paying for drugs, we have to also think about what the broader societal benefits are.”—citizen/pharmacist or allied health professionals group “I do agree with her about the (added) benefits and the QALY. You do have to put them side by side.”—patient/caregiver group “What I would really like is to have a measurement that was QALY with the numerator of this societal return of investment so that we can see what the societal gain is and the gain that patients bring together. And maybe these kinds of forums where you get feedback will lead to some better metrics that cover both of those things.”—citizen/pharmacist or allied health professionals group

Information should be accessible, simple, and understandable to the public.
“I like the QALY because of the simplicity because it covers the really two big important things that we care about: quality of life and how long people live.”—citizen/pharmacist or allied health professionals group “I also like it (the QALY) because it actually seems to be more publicly accessible that somebody like me maybe could find out from someone like you where the when the numbers came from.”—citizen/pharmacist or allied health professionals group “I prefer the added benefit, and then the clinical benefit rating, the idea of a category such as major additional benefit or somewhat additional or I guess a better categorization than a numerical just the number 14.2 versus 13, which is not a necessarily game changer.”—patient/caregiver group

Table 3 summarizes the top health gain measures and selected quotes discussing them. We categorized the quotes according to their corresponding themes and representing perspectives. These quotes offer additional insights into the ranking results. Specifically, participants provided information on how the aspects they valued in health gain measures related to the QALY, MCDA, and clinical benefit rating. Furthermore, we added a “thumbs-up” or “thumbs-down” icon to each quote to illustrate whether it supported or opposed the measure (see Appendix S4 for full themes and quotes from breakout and combined deliberations).

**Table 3.** Selected quotes for the top 3 health gain measures.

Measure	Group 1: patients and caregivers	Group 2: citizen, pharmacist, or allied health professionals
QALY	Major theme: Information should be accessible, simple, and understandable to the public.	
	“Speaking for myself, I have a much better understanding of QALY and DALY, but I have a much less understanding of MCDA.” 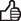	“I like the QALY because of the simplicity because it covers the really two big important things that we care about: quality of life and how long people live.” 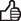 “I also like it (the QALY) because it actually seems to be more publicly accessible that somebody like me maybe could find out from someone like you where the when the numbers came.” 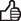
	Major theme: Patient input is important for determining values in population-based health gain measures.	
	“The reason I feel like it (the QALY) may be a little skewed, is because perspective on what is quality of life. I cared for my mom for a long time, basically her whole life, and she lived with a lifelong disability, which everyone would be like, that's a disability. But for her, she overcame so many of those things that people thought were hindering her. She found so many unique ways to get over that.” 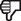	
	Minor theme: Want to minimize subjectivity and maximize objectivity.	
		“It just seems to me that the QALY is the most objective. Although I'm not really happy that groups of patients and community people are deciding that hearing loss is like 80% of life. That's a little less objective, but it does seem like there are instances where that is the way to go.” 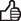
	Minor theme: Want to prioritize measures with more years of use.	
	“[person 1] With the ones we were going through today. HYT and the evLY, I really don't want that one because when I look at the limitations and the challenges, I feel like the QALY is more ahead of it. [person 2] It's (QALY) been used more in practice. It has a longer track record.” 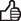	
MCDA	Major theme: Information should be accessible, simple, and understandable to the public.	
	“Speaking for myself, I have a much better understanding of QALY and DALY, but I have a much less understanding of MCDA.” 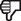	
	Major theme: Patient input is important for determining values in population-based health gain measures.	
		“As a rehabilitation specialist, I am thinking that if you (health professionals) choose anything that measures disability level, the people that I work with (patients) are not going to be happy because they think it's discriminatory. I prefer the one (health gain measure) that the patient will choose. So, (I prefer) the MCDA or whatever. The patient will choose the one that he or she prefers.” 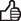 “But a group of patients specific to a condition. A group of psoriasis patients, a group of pancreatic cancer patients, or a group of diabetic patients could make a decision (with MCDA) that could be representative of them with input from clinical guidelines and prescribers. And the medical background says that, and these are the drugs we think are the most beneficial to us based on what we value.” 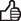
	Minor theme: Want to minimize subjectivity and maximize objectivity.	
	“I feel like the one you just did (MCDA) is very subjective, and I don’t think it will work for the masses.” 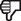	
Clinical benefit rating	Major theme: Information should be accessible, simple, and understandable to the public.	
	“I prefer the added benefit, and then the clinical benefit rating, the idea of a category such as major additional benefit or somewhat additional or I guess a better categorization than a numerical just the number 14.2 versus 13, which is not a necessarily game changer.” 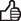	

### Dashboard results

The study team developed a draft dashboard following the first meeting (see Appendix Figures S1 and S2). Here, we highlight 3 important pieces of feedback from the participants during discussion of this dashboard.

First, the participants thought the descriptions and nuances on the draft dashboard were too detailed. Consistent with major theme number 3 highlighted above, they wanted the dashboard to be succinct and self-explanatory. They preferred reporting a final number to deliver a clear message, rather than reporting multiple numbers to reflect the steps and nuances involved in the complex estimation of the value of health gain measures. Second, the participants urged that the colors should be “colorblind safe” to allow for widespread use. Participants discussed the best color to present specific information, but the group did not reach consensus. As a result, the study team made a final judgment on the colorblind safe palette. Third, participants conveyed their preferences for displaying the QALY. It is common for researchers to present the QALY data in a graph with 2 axes. The horizontal axis represents the LYs, and the vertical axis represents utility or quality of life. Researchers typically prefer this because it illustrates the impact of treatment on both length and quality of life simultaneously. In contrast, our participants preferred a bar graph that summarizes the QALYs rather than showing the nuances in changes among LYs and quality of life, indicating a preference for a simpler presentation with supporting text to describe the details.

**Figure 1.**
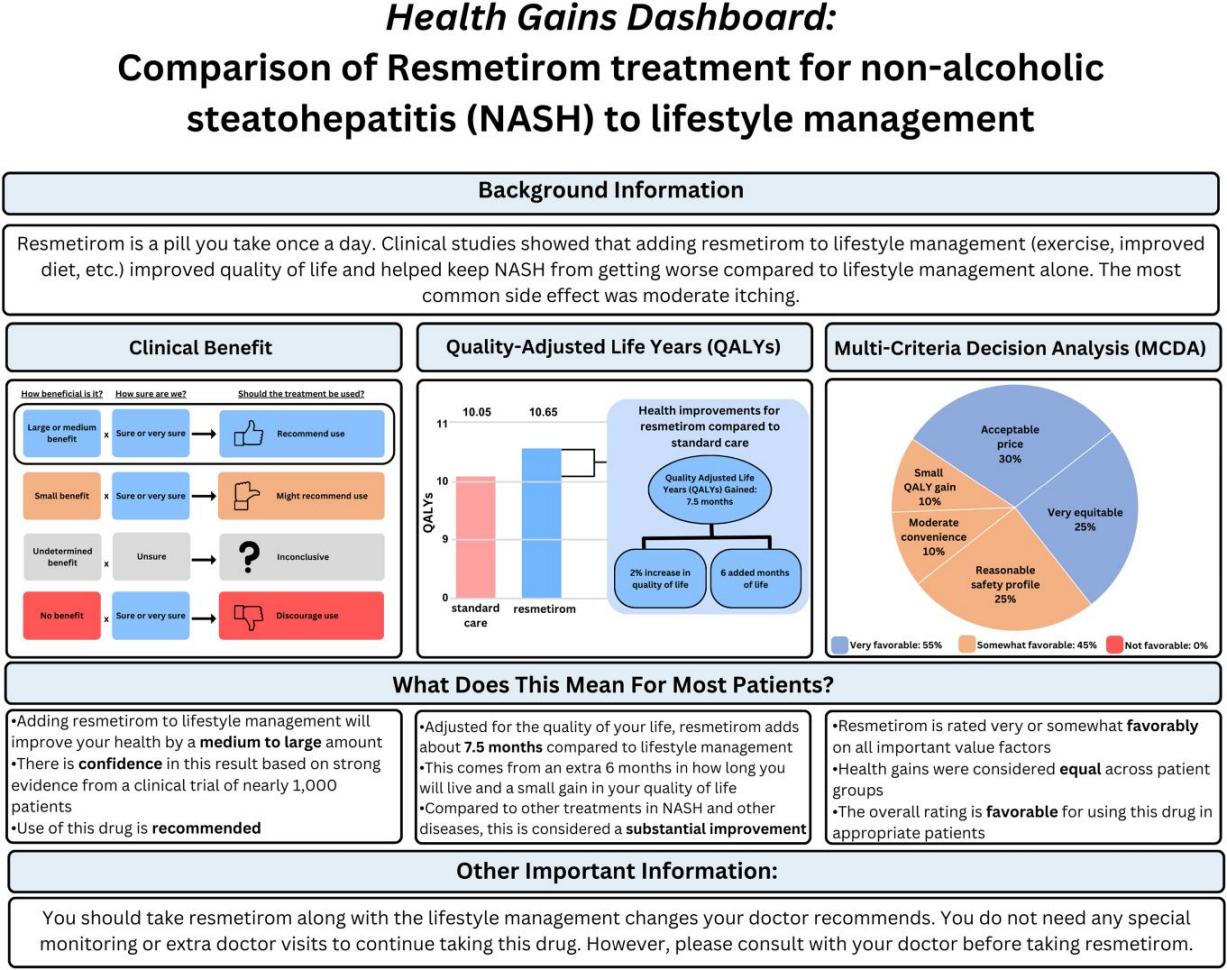
Final dashboard for displaying health gain measures.

Figure 1 shows the revised dashboard incorporating participants’ feedback. The final dashboard has 4 sections: background information, health gains illustrated graphically and descriptively, and other important information. At the top, the background information section describes the intervention, the comparator, and the health condition of interest. In the middle, the health gain illustration sections visually show health gains using the QALY, clinical benefit rating, and MCDA. Correspondingly, texts below each graph elaborate on the information and significance of the health gain measures. At the bottom, the other important information section offers useful details not covered in the preceding sections.

## Discussion

To the best of our knowledge, this is the first study to use public deliberation to understand stakeholder and community member preferences for health gain measures. The results reported in this study capture preferences driven by participants’ experience and values. Furthermore, this is the first study to develop a dashboard prototype that is based on stakeholder input on how to deliver key messages using these measures.

The QALY emerged as the most favorable health gain measure in this study because it quantifies quality of life and length of life in a simple way. This may be surprising to researchers and policymakers in the field given the history of opposition by certain groups to this measure, including patient advocacy groups, who consider the QALY to be discriminatory toward the disabled and severely ill.^18-20^ Additionally, certain Federal programs in the United States are prohibited from using the QALY due to these concerns about discrimation.^21^ Anti-QALY sentiment has also been fueled by heavy lobbying from the life sciences industry, and many of the patient groups arguing against use of the QALY are financially supported by industry.^22^ However, other international organizations in countries where the industry lobby is not as powerful, such as the National Institute for Health and Care Excellence in the United Kingdom and the Canadian Agency for Drugs and Technologies in Health, have been using the QALY to make policy decisions for decades.^23,24^ In a 2021 study, the QALY was the most recommended health gain measure used in national healthcare economic evaluation guidelines internationally.^25^

The most discussed theme in our analysis was “Patient input is important for deriving values in population-based health gain measures.” This finding contrasts with most HTA guidelines, which suggest that preferences used in calculating QALYs should be based on the general population rather than individual patients.^26^ Most HTA bodies favor population-based preferences due to their operation within publicly funded universal healthcare systems.^27^ However, the setting and context of this study are important to note, as the decentralized US healthcare system and the culture of American individualism mean that many individuals in the United States prioritize individual benefits over maximizing social welfare.^28^ These values likely contributed to the themes we observed in our study.

It is notable that the QALY ranked highest in our study, despite some opposition to this measure in the United States. Future research should include public deliberation sessions focused solely on stakeholders’ acceptance of the QALY, building on our study and further addressing the ethical concerns and controversies surrounding it. These sessions would benefit from the involvement of ethicists and methodologists who can engage the community in discussions about the advantages and disadvantages of the QALY and its application in various case studies. This approach would provide valuable insights into how different scenarios shape community perspectives and could further explore the acceptability of the QALY and similar measures in the United States.

Clinical benefit rating ranked second in the final poll, and despite the lack of explicit discussion around this measure, it is likely that participants favored this measure because it is simple and understandable. Indeed, a final letter grade derived from the categorization of health benefits and confidence in the evidence makes the recommendation clear and easy to incorporate. For example, the USPSTF uses a clinical benefit rating to disseminate its recommendations regarding the adoption of preventative tests and services to a diverse audience, including patients, researchers, primary care clinicians, advocacy groups, and policymakers.^15^ However, while the results from clinical benefit ratings are easy to interpret, comparing the results between disease areas is challenging. For instance, it is unclear how limited resources should be allocated when 2 interventions receive the highest rating from such an approach.^29^

Participants also favored MCDA as a health gain measure, primarily due to its flexibility in incorporating multiple key factors that are important to individuals, such as side effects, quality of life, and cost. One benefit of MCDA is that it can incorporate quantitative and qualitative data to help policymakers with healthcare decisions.^30^ Furthermore, MCDA may offer a platform for integrating emerging novel value elements, such as real option value, or the value of hope, which are difficult to quantify and often overlooked in traditional HTA.^31^ Interestingly, there are government agencies adopting or piloting MCDA, and studies have shown an increasing trend in applying MCDA for HTA studies.^13,32^ On the other hand, despite recognizing the capability to take into account multiple factors, patient and caregiver stakeholders expressed concerns regarding the various potential problems with MCDA. For example, there is no consensus on criteria selection and weights for each criterion in healthcare decision-making. That is, different stakeholder groups might select distinct criteria and weights for the same issue, leading to different conclusions. Therefore, MCDA users need to be aware of potential biases arising from criteria selection and weighting, including representativeness, overweighting, and double counting.^30^

Participants showed varying perspectives and preferences. The discordance in ranking was expected, as previous research has found that patients and healthcare providers often have differing preferences in healthcare.^33^ We hypothesized at the start of the study that all groups would have unique preferences shaped by their background and experience. Qualitative analysis of these deliberations helped us understand the discordance in quantitative rankings among stakeholders.

## Limitations

This study has several limitations. First, participants represented a limited set of stakeholder perspectives. There are other important stakeholders not included in this study, such as the pharmaceutical industry, insurance, and government representatives. However, engaging broader perspectives in a larger group can potentially lose the in-depth deliberation we had with a smaller set of participants. Second, participants were those who completed our initial survey questions, had the time to attend in-person sessions, and had the ability to travel to attend these discussions. They were therefore a subset of the pool of potentially interested individuals, and the extent to which they accurately represent the larger pool is unknown. Third, our participants are primarily from the greater Boston area. Stakeholders from other locations may have different opinions on the health gain measures we deliberated on and may have produced different ranking results. Fourth, the study team's perspective on the health gain measures may have biased the results. These biases could stem from the development of educational materials, the delivery of educational sessions, and the clarification of questions raised by participants. Despite this, our approach facilitated participants expressing their opinions openly and prevented the deliberations from being skewed in any particular direction.^34^ Fifth, the study team did not specifically assess participants’ understanding of these measures. However, each participant had multiple opportunities to ask clarifying questions, actively contributed to the discussion, and deliberated on the important elements of each health gain measure. All participants also actively contributed to the development of the dashboard that was designed to present the top-ranked health gain measures in a way that was accessible to a lay audience. Sixth, we did not recruit a representative sample within each group with respect to age, sex, education, and income due to our small sample size. A larger sample with a greater representation within groups may have produced different results. Future research on this topic should explore other deliberative approaches that may facilitate larger, more representative, groups of individuals.

## Conclusion

This deliberation exercise explored the pros and cons of 9 health gain measures with a multistakeholder group. Participants highlighted the importance of patient input in developing health gain measures, the benefit of using multiple complementary measures to evaluate societal benefits, and the need for information to be accessible, simple, and understandable. The QALY, a measure of quality and length of life, was the group's most favored health gain measure, followed by clinical benefit rating and MCDA. The selection of the QALY as the preferred measure suggests that policymakers may want to revisit previous judgments and stereotypes about these measures. Our discussions illustrated that these measures can be integrated into a user-friendly “dashboard” that contains succinct and straightforward information that helped our participants understand the health gain measures. This template can be modified to be used more broadly to help convey health gain information and promote informed decision-making.

## Supplementary Material

qxae111_Supplementary_Data
