# Supplementary material for: Public deliberation on health gain measures
Source: Health Aff Sch. 2024 Sep 9;2(9):qxae111. doi: 10.1093/haschl/qxae111 (PMC11412319; doi:10.1093/haschl/qxae111)
Supplement: qxae111_Supplementary_Data [file qxae111_supplementary_data.zip › Appendix 1 - Screening questions.docx]

Contact Information

First Name

Last Name

Phone

Email Address

How would you prefer to be contacted?

Phone

Email

We will host the first full-day meeting between Sep 18th and Sep 24th.
Please indicate your preferred availability for the first meeting. (Check all that apply)

I am not available on any days during this date range

I am available any weekday within this date range

I am available any weekend day within this date range

I am available only on specific date(s):_ _____ (show text box when they check this option)

We will host the second half-day meeting between Oct 25th and Nov 1st.
Please indicate your preferred availability for the second meeting. (Check all that apply)
 I am not available on any days during this date range

I am available any weekday within this date range

I am available any weekend day within this date range

I am available only on specific date(s):_ _____ (show text box when they check this option)

Would you prefer the morning or afternoon for the second half-day meeting?

Morning

Afternoon

How old are you?

17 years or younger

18 - 24 years

25 - 34 years

35 - 44 years

45 - 54 years

55 - 64 years

65 - 74 years

75 years or older

Prefer not to say

What gender do you most identify with?

Female

Male

Genderqueer/Gender nonconforming/Non-binary

Other, please specify

Which race best describes you? (Please choose only one.)

American Indian or Alaskan Native

Asian

Black or African American

White

Native Hawaiian or Other Pacific Islander

Multiracial

Other, please specify

Prefer not to say

Are you of Hispanic or Latino origin?

Yes

No

Prefer not to say

What is the highest level of school you have completed?

Less than a high school diploma

High school diploma or equivalent (e.g., GED)

Some college credit, but no degree

Associate’s degree

Bachelor’s degree

Graduate degree

Prefer not to say

Other, please explain (text)

Please specify the highest level of school you have completed

What is your total household income?

Less than $20,000

$20,000 to $39,999

$40,000 to $79,999

$80,000 to $119,999

$120,000 to $159,999

$160,000 to $199,999

$200,000 or more

Prefer not to say

Are you a native English speaker?

Yes

No

If no: How would you rate your level of proficiency in English?

Reading:

(drop-down list:

(1) Elementary: can read very simple written material in English

(2) Working: can read most factual material written material in English

(3) Advanced Professional: can read fluently and accurately all styles and forms of English pertinent to professional needs)

Listening: (drop-down list from (1) Elementary to (3) Advanced Professional)

(drop-down list:

(1)Elementary: can understand enough English for basic survival needs and minimum courtesy and travel requirements

(2)Working: can understand enough English for routine social demands and limited job requirements

(3) Advanced Professional: can understand all forms and styles of in English pertinent to professional needs)

Speaking: (drop-down list from (1) Elementary to (3) Advanced Professional)

(drop-down list:

(1)Elementary: can maintain very simple face-to-face English conversations on familiar topics

(2)Working: can speak enough English to satisfy routine social demands and limited work requirements.

(3) Advanced Professional: can speak English fluently and accurately on all levels normally pertinent to professional needs.)

We will ask individuals involved in this project to provide their perspectives based on their experience in the categories below. What is the primary perspective you would like to contribute to this project? If you fall into more than one category, please select the perspective that is most meaningful to you.

Individual with or at risk of a specific health condition (for example, individuals who are living with illness, and/or those who directly experience (or have previously experienced) the benefits or harms associated with medical care)

Patient representative from a patient advocacy group

Caregiver (individual who cares for a person living with a health condition)

Caregiver representative from an advocacy group

Citizen (a member of the general public or a representative from a civil society group, such as labor unions and professional associations)

Clinician (for example, physician, nurse, dentist)

Pharmacist or allied health professional (clinical practitioners that work across diverse settings and fields)

Other (please specify)

Do you have any experience with or knowledge of approaches used to value improvements in health (e.g., quality-adjusted life year, or health years in total)?

Yes

No

If yes: How would you rate your level of prior knowledge or exposure to these approaches?

High (I am very familiar with these concepts and approaches)

Medium

Low (I have seen or heard about these concepts and approaches but could not explain them)

How good are you at working with fractions?

Dropdown list: Not good at all, 1” to “Extremely Good, 6

How good are you at figuring out how much a shirt will cost if it is 25% off?

Dropdown list: Not good at all, 1” to “Extremely Good, 6

How often do you find numerical information to be useful?

Dropdown list: Never, 1” to “Very Often, 6

--End of screening--
