# Supplementary material for: Public deliberation on health gain measures
Source: Health Aff Sch. 2024 Sep 9;2(9):qxae111. doi: 10.1093/haschl/qxae111 (PMC11412319; doi:10.1093/haschl/qxae111)
Supplement: qxae111_Supplementary_Data [file qxae111_supplementary_data.zip › Appendix 2 - Educational materials.docx]

**Educational Material Guide**

Thank you for participating in the public deliberation on the best ways to measure health improvements. Below, you will find educational materials that will help familiarize you with some ways that researchers measure health improvements.

Please note that a complete understanding of all the health gain measures is not required for the meeting. The purpose of this educational material is to present high level concepts.

We kindly request that you review these materials and come prepared before the first in-person meeting. Please feel free to ask any questions you may have. Before the discussion begins, we will elaborate on these health gain measures and provide answers to your questions. Additionally, you can suggest a way to measure health improvement that is not on the list. If you do so, we ask that you provide links to educational materials and send the link to us before the first meeting. We will share the link and it can help all participants understand the measure and its application.

Once again, we sincerely appreciate your participation, and we eagerly look forward to meeting you in person.

**Educational Material Instructions**

It will take approximately 1.5 hours to review all the educational materials. You will be able to access the educational materials by clicking the word “Link” in the third column in both Windows and Mac systems.

Here are the steps for each operating system:

Windows:

1. Locate the link you want to follow.

2. Position your cursor over the link. It should change to a hand cursor or show an underline.

3. “control+click” once to follow it. This should open the link in your default web browser.

Mac:

1. Find the link you wish to follow.

2. Place your cursor over the link. It should change to a hand cursor or show an underline.

3. “Cmd+Click” once to follow it. This action will open the link in your default web browser.

| Health Gain Measure | Definition and note | Link to educational materials |
| --- | --- | --- |
| 1. LY (Life Year) | LY= How much longer you would be expected to live (your life expectancy) given your age, current health, and other factors | Self-explanatory. |
| 1. QALY (Quality-Adjusted Life Year) | QALY = Your life expectancy, adjusted by the quality of life you experience during those years | [Link](https://lrs.smartbuilder.com/l/o754jrapoo1i): A 10-minute narrated presentation that describes that QALY, and some pros and cons of the QALY. Use the bar on the top right of the presentation to advance slides. |
| 1. evLY (Equal Value Life Years) | evLY = Like the QALY, except that years of life added as a result of treatment are adjusted by the quality of life of an average healthy person in the US population, and not the quality of life of the patient population being studied. | [Link](https://youtu.be/lKwetP2IXeE): A 2-minute video providing high level introduction. Need to know the concept of QALY first. |
| 1. DALY (Disability-Adjusted Life Year) | DALY = a measure of how much burden a disease causes, based on the number of years lost from sickness, disability, and/or early death. Researchers can measure health gain by measuring how many DALYs can be avoided with a new treatment versus an old treatment. | [Link](https://youtu.be/Exce4gy7aOk): A 9-minute video that explains what is a DALY, provides an overview of DALYs averted (i.e. DALYs that are avoided with a new treatment), and compares the DALY with the QALY. |
| 1. VSL (Value of a Statistical Life) | VSL = A measure of how much money you would be willing to pay to avoid a small chance of dying. | [Link](https://www.epa.gov/environmental-economics/mortality-risk-valuation#whatisvsl): A page that explains how the Environmental Protection Agency measures VSL in economic analyses. See paragraph titled “What does it mean to place a value on life?” |
| 1. Added Benefit | A classification of how much better (or worse) a new treatment is at improving your health vs. one or more alternatives. An intervention’s “added benefit” can be classified into 6 levels, as described in the educational materials. The classification is typically made based on the judgement of members of a health technology assessment organization, and the analyses they conduct relevant to the intervention. | [Link](https://app.box.com/s/bxw1y63yg5nfd8nuog8957wzp3632kza): Describes the added benefit framework in Germany. See page 4 titled 'What Is Added Benefit?,' page 6 titled 'Discussion,' and page 7 titled 'Relevance to U.S. Health Reform Efforts'  [Link](https://app.box.com/s/zm0nqmp338exnk5i51ls726g2200tah6): Compares Germany and France. Focus on table 1 which summarizes the classification systems in each country |
| 1. Clinical Benefit Rating | The same as added benefit, but also considers how confident you are in the judgment (how strong is the evidence?) | [Link](https://www.youtube.com/watch?v=goJxhYxm2ZU): A 7-minute video that explains the rating system and provides examples with case studies in US  [Link](https://blog.prevounce.com/6-things-to-know-about-the-preventative-services-task-force) (optional): A blog post that discusses the interaction between the Affordable Care Act (ACA) and the U.S. Department of Health and Human Services (HHS). |
| 1. MCDA (Multi-Criteria Decision Analysis) | A system that assigns weights to certain health, social, and other factors that might be important in judging the value of a treatment | [Link](https://youtu.be/7OoKJHvsUbo): 4 minutes video on how MCDA works using graphs.  [Link](https://app.box.com/s/c98oqfduyue1u0gpf50r1oy0ln7ag07f) (optional): Focus on p.14-p16 for MCDA introduction. |
| 1. SROI (Social Return On Investment) | Like MCDA, but expresses both the value factors in $$$ amounts | [Link](https://youtu.be/og4m8BqxWL0): A video (from 5:21 to 11:45) that describes an example used in Canada.  [Link](https://app.box.com/s/rbrc2hyh9epb8pagwu8km1nwqorocv8j) (optional): Information on the social values |
| 1. (optional). Additional measurement not listed above: |  | If you have additional measurement that is not listed above, please provide links to educational materials and send the link to us before the first meeting |
