# Supplementary material for: Public deliberation on health gain measures
Source: Health Aff Sch. 2024 Sep 9;2(9):qxae111. doi: 10.1093/haschl/qxae111 (PMC11412319; doi:10.1093/haschl/qxae111)
Supplement: qxae111_Supplementary_Data [file qxae111_supplementary_data.zip › Appendix 3 - Participant characteristics_revise (clean).docx]

# Appendix 3- Participant characteristics (n=20)

|  | Number of participants |
| --- | --- |
| **Primary perspective** | |
| Patient | 5 |
| Patient representative from a patient advocacy group | 3 |
| Caregiver | 3 |
| Caregiver representatives from advocacy groups | 1 |
| Citizen | 4 |
| Pharmacist or allied health professional | 4 |
| **Sex** | |
| Female | 12 |
| Male | 8 |
| **Age** | |
| 25 - 34 years | 6 |
| 35 - 44 years | 1 |
| 45 - 54 years | 3 |
| 55 - 64 years | 4 |
| 65 - 74 years | 5 |
| 75 years or older | 1 |
| **Race** | |
| White | 11 |
| Asian | 2 |
| Black or African American | 5 |
| Multiracial | 1 |
| Other | 1 |
| **Highest level of education** | |
| High school diploma or equivalent (e.g., GED) | 1 |
| Some college credit, but no degree | 3 |
| Associate's degree | 2 |
| Bachelor's degree | 2 |
| Graduate degree | 12 |
| **Total household income** | |
| Less than $20,000 | 4 |
| $20,000 to $39,999 | 3 |
| $40,000 to $79,999 | 3 |
| $80,000 to $119,999 | 2 |
| $120,000 to $159,999 | 2 |
| $160,000 to $199,999 | 2 |
| $200,000 or more | 2 |
| Prefer not to say | 2 |
| **Literacy test** | |
| **Are you a native English speaker?** | |
| Yes | 18 |
| Did not answer | 1 |
| No | 1 |
| If no: How would you rate your level of proficiency in English? |  |
| Reading: | Advanced Professional* |
| Listening: | Advanced Professional |
| Speaking: | Advanced Professional |
| **Numeracy test** | |
| **How good are you at working with fractions?** | |
| 1, not good at all | 0 |
| 2 | 0 |
| 3 | 1 |
| 4 | 6 |
| 5 | 7 |
| 6, extremely good | 6 |
| **How good are you at figuring out how much a shirt will cost if it is 25% off?** | |
| 1, not good at all | 0 |
| 2 | 0 |
| 3 | 2 |
| 4 | 2 |
| 5 | 6 |
| 6, extremely good | 9 |
| Did not answer | 1 |
| **How often do you find numerical information to be useful?** | |
| 1, never | 0 |
| 2 | 0 |
| 3 | 0 |
| 4 | 0 |
| 5 | 0 |
| 6, very often | 11 |
| Did not answer | 9 |

* Description of an advanced professional in the survey question: can understand all forms and styles of English pertinent to professional needs. See Appendix 1 (Screening questions).
