# Supplementary material for: Public deliberation on health gain measures
Source: Health Aff Sch. 2024 Sep 9;2(9):qxae111. doi: 10.1093/haschl/qxae111 (PMC11412319; doi:10.1093/haschl/qxae111)
Supplement: qxae111_Supplementary_Data [file qxae111_supplementary_data.zip › Appendix 4 - Full themes and quotes.docx]

| Major themes | Count | Group 1: patients and caregivers | Group 2: citizen, pharmacist or allied health professionals | Combined group (second deliberation) |
| --- | --- | --- | --- | --- |
| Patient input is important for deriving values in population-based health gain measures. | 12 | "I wouldn't trust somebody who never had any experience with disabilities or has never been disabled to be able to weigh those things (health gain measures) (in a way) that I would feel comfortable with."  "I went through this (disconnect between patient and community values) towards my mom's later part of life. She was bed-bound. And for some people, they used to be like, what type of life is bed-bound? But for her, it was still being able to see her grandkids, still being able to laugh, have fun, and eat. Nothing else stopped besides the fact that she couldn't walk. So, for a lot of people, that's not a good life, but for her, that's a good life."  "The reason I feel like it (the QALY) may be a little skewed, is because perspective on what is quality of life.   I cared for my mom for a long time, basically her whole life, and she lived with a lifelong disability, which everyone would be like, that's a disability. But for her, she overcame so many of those things that people thought were hindering her. She found so many unique ways to get over that."  "I kind of went through this (disconnect between patient and community values) towards my mom's later part of life. She was bed-bound. And for some people, they used to be like, oh, well, what type of life is bed-bound? But for her, it was still being able to see her grandkids, still being able to laugh, have fun, and eat. Nothing else stopped besides the fact that she couldn't walk. So, for a lot of people, that's not a good life, but for her, that's a good life."  "I don't think there's a good solution because different people value different things, and in the context of their life, it makes perfect sense. But when you look at it from the outside, you say, well, why would anybody want to live like that? " | "The ones that use the patient input are slightly better because they're [the patients are] deciding for themselves the impact on their own lives, which personally feels much better."  "It feels so important to have like the actual people whose actual lives are going to be impacted, involved in the decision making on a really high level"  "As a rehabilitation specialist, I am thinking that if you (health professionals) choose anything that measures disability level, the people that I work with (patients) are not going to be happy because they think it's discriminatory. I prefer the one (health gain measure) that the patient will choose. So, (I prefer) the MCDA or whatever. The patient will choose the one that he or she prefers."  "A group of patients specific to a condition. A group of psoriasis patients, a group of pancreatic cancer patients, or a group of diabetic patients could make a decision (with MCDA) that could be representative of them with input from clinical guidelines and prescribers. And the medical background says that, and these are the drugs we think are the most beneficial to us based on what we value."  "If the research preference is to have those weights be derived based on general community preferences, someone in the disability community could defensively say that is an ablest approach. You're telling me how it feels… to be disabled."  "I would argue that you could flip it through. Get overall community perspectives and then have your specific patient population or the specific patient that you're measuring review the results from the community. And say yes, this fits my perspective. No, this doesn't fit my perspective, et cetera, and validate it in that sense."  "I have these conversations with patients all the time about their preferences, how they are weighing their decisions, and why. We need to find ways to collect and aggregate this data, using it as a framework for decision-making rather than relying on arbitrary criteria." |  |
| Need multiple complementary measures to see full range of societal benefits. | 8 | "I do agree with her about the (added) benefits and the QALY. You do have to put them side by side." | "Most of the measurements are focused on patients, and the patients’ immediate surrounding in their care group is their family. But if you're thinking about policy and how much we should be paying for drugs, we have to also think about what the broader societal benefits are."  "What I would really like is to have a measurement that was QALY with the numerator of this societal return of investment so that we can see what the societal gain is and the gain that patients bring together. And maybe these kinds of forums where you get feedback will lead to some better metrics that cover both of those things."  "The evLY weighs so heavily just life years. That's good for some things, and for some drugs, that's insanely important, and for some, it is the least important thing about it." | "I particularly like devaluing the ones that include life year as a heavily weighted part of the calculation because that's not necessarily the most important thing happening. And I worry that it would devalue drugs that have many other positive benefits for general treatment."  "I put QALY first and MCDA second. I thought both of them serve different kinds of objectives and thought the combination would be a good one.”  "I had the clinical benefit rating and the added benefit (at the top of my ranking). Parts of those things should be to be added into the equation of QALY."  "That's why we decided that when we picked the quality adjustment of life and the one after that (clinical benefit rating) is because we figured we could combine the two or put them side by side together, which is probably close to what your MCDA was." |
| Information should be accessible, simple, and understandable to the public. | 4 | "I prefer the added benefit, and then the clinical benefit rating, the idea of a category such as major additional benefit or somewhat additional or I guess a better categorization than a numerical just the number 14.2 versus 13, which is not a necessarily game changer."  "Speaking for myself, I have a much better understanding of QALY and DALY, but I have a much less understanding of MCDA." | "I like the QALY because of the simplicity because it covers the really two big important things that we care about: quality of life and how long people live."  "I also like it (the QALY) because it actually seems to be more publicly accessible that somebody like me maybe could find out from someone like you where the when the numbers came from." |  |
| Minor themes | Count | Group 1: patients and caregivers | Group 2: citizen, pharmacist or allied health professionals | Combined group (second deliberation) |
| Values should be obtained from individual patients to inform their own decision making | 4 | "I think patients have to be the ones that make these decisions because I've dealt with a lot of doctors who even would say, well, that's not a good quality of life. I've even had doctors put my mom on hospice and told me what she was gonna do, and my mom did the complete opposite. "  "I don't think there's a good solution because different people value different things, and in the context of their life, it makes perfect sense. But when you look at it from the outside, you say, well, why would anybody want to live like that? " | "Pancreatic cancer usually has like six months to live, but they (my friend and my friend's mom) just want to stand. You know, everyone has different goals, right? (…) So I really do think if you're gonna measure health outcomes, you should have the patient weigh what they want like (…) every patient does value something differently." | "We liked MCDA because you could take everything that was numbers and add in what patients were experiencing to make a decision of your own.” |
| Community input can be used alongside with patient input to determine values used in population based health gain measure | 3 |  | "What about a community group whose charge is to review the information (gathered) from disabled people?"  "Get overall community perspectives and then have your specific patient population or the specific patient that you're measuring review the results from the community. And say yes, this fits my perspective. No, this doesn't fit my perspective, et cetera, and validate it in that sense."    "Then you got to look for a different group (for health gains measure values), make that community group subject to approval by the disabled group." |  |
| Equity is important | 2 |  |  | "I particularly like MCDA because it can include some of those dimensions of equity (…) As I'm representing patient groups today, I felt that the issue of equity, the ability to serve people who are less advantaged, who have language difficulties, and who have difficulty understanding the medical systems in this country, would benefit from something that is more comprehensive like MCDA."  "I do not like it (MCDA) at all. That would probably be one of the least. I don't trust the scale. I would have to really thoroughly look through the people who would be on that board of making the weighted thing because I feel like it's subjective, and I don't know if that would give equity." |
| Like flexibility | 2 |  |  | "In my mind, MCDA was allowing us to combine a whole bunch of things and make it whatever it needed to be for the situation we were given. We could use a lot of criteria and a lot of patient perspectives and expert unions."  "We liked MCDA because you could take everything that was numbers and add in what patients were experiencing to make a decision of your own.” |
| Want to minimize subjectivity and maximize objectivity | 2 | "I feel like the one you just did (MCDA) is very subjective, and I don't think it will work for the masses." | "It just seems to me that the QALY is the most objective. Although I'm not really happy that groups of patients and community people are deciding that hearing loss is like 80% of life. That's a little less objective, but it does seem like there are instances where that is the way to go." |  |
| Want to prioritize measures with more years of use | 1 | "[person 1] With the ones we were going through today. HYT and the evLY, I really don't want that one because when I look at the limitations and the challenges, I feel like the QALY is more ahead of it. [person 2] It's (QALY) been used more in practice. It has a longer track record." |  |  |
