# Supplementary figures and images for: Public deliberation on health gain measures
Source: Health Aff Sch. 2024 Sep 9;2(9):qxae111. doi: 10.1093/haschl/qxae111 (PMC11412319; doi:10.1093/haschl/qxae111)

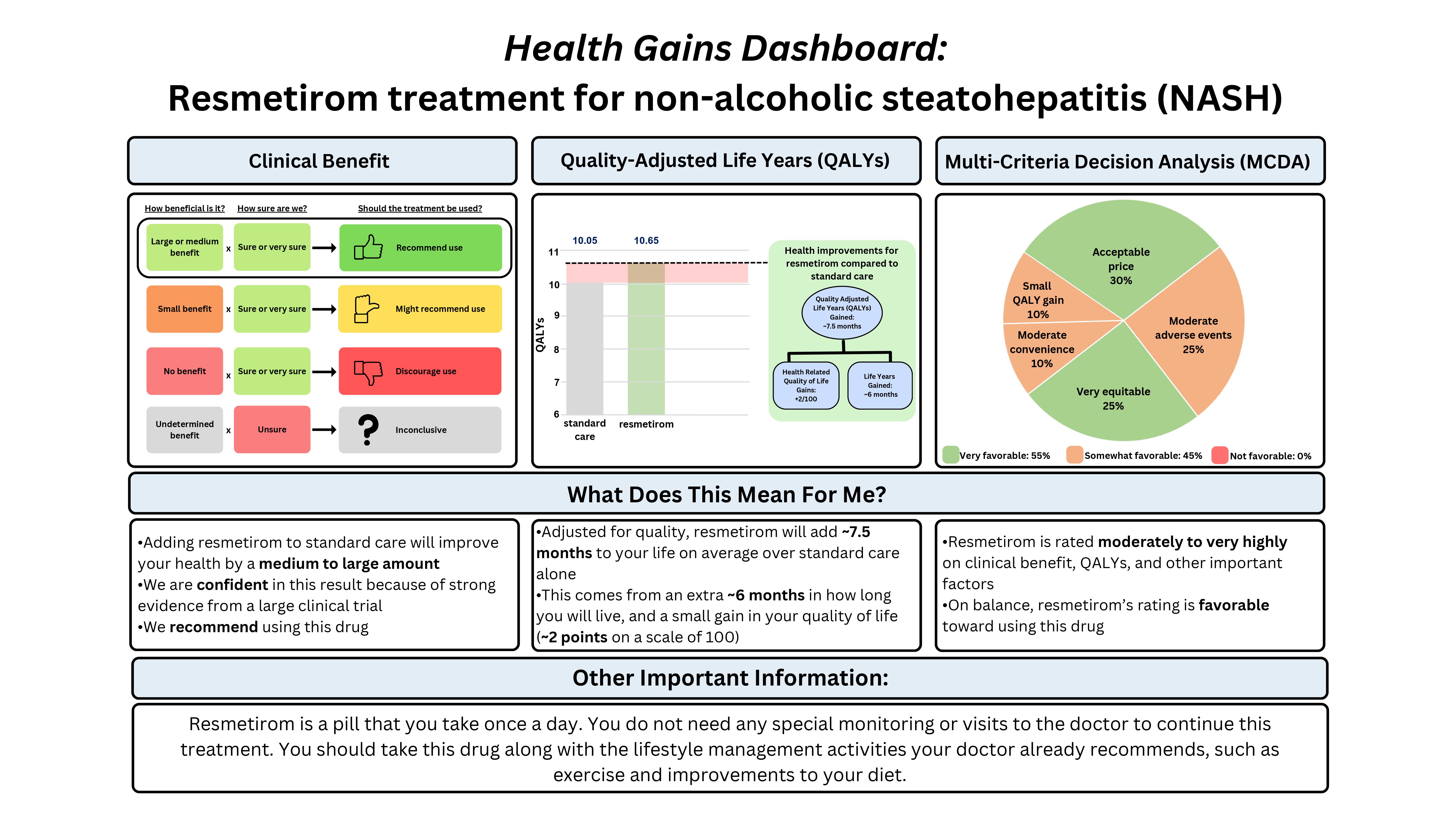

Supplement: qxae111_Supplementary_Data [file qxae111_supplementary_data.zip › Appendix Figure 2.jpg]

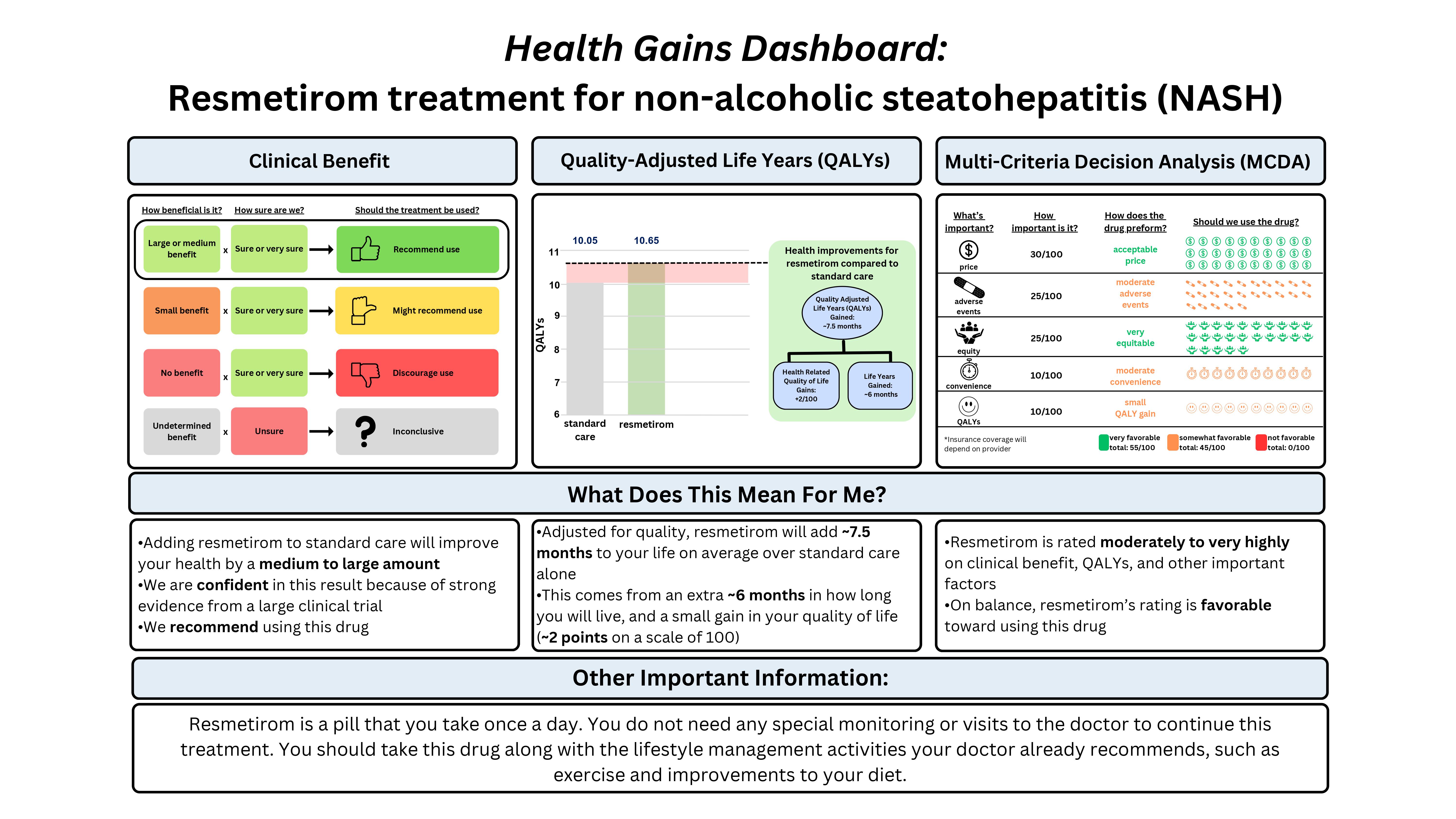

Supplement: qxae111_Supplementary_Data [file qxae111_supplementary_data.zip › Appendix Figure 1.jpg]
